# Strain variation in early innate cytokine induction by *Plasmodium falciparum*

**DOI:** 10.1111/j.1365-3024.2010.01225.x

**Published:** 2010-07

**Authors:** R A CORRIGAN, J A ROWE

**Affiliations:** Centre for Immunity, Infection and Evolution, Institute of Immunology and Infection Research, School of Biological Sciences, University of EdinburghEdinburgh, UK

**Keywords:** innate immunity, interferonγ, Malaria, virulence

## Abstract

Previous work has shown that human donors vary in the magnitude and pattern of cytokines induced when peripheral blood mononuclear cells (PBMCs) are co-cultured with *Plasmodium falciparum–*infected erythrocytes. Whether *P. falciparum* strains vary in their ability to induce cytokines has not been studied in detail and is an important question, because variation in cytokine induction could affect parasite virulence and patterns of clinical disease. We investigated the early inflammatory cytokine response to four *P. falciparum* laboratory strains and five field isolates. Initial studies showed that parasite strain, parasitaemia and PBMC donor all had significant effects on the magnitude of pro-inflammatory cytokine responses (IFN-γ, GM-CSF, IL-1β, TNF-α, IL-6, *P* < 0·005 in all cases). However, we noticed that the most highly inducing parasite strain consistently reached schizont rupture more rapidly than the other strains. When timing of schizont rupture was taken into account, parasite strains no longer differed in their cytokine induction (*P* = 0·383), although donor effects remained significant (*P* < 0·001). These data do not support the hypothesis that *P. falciparum* strains vary in induction of early innate cytokine responses from PBMCs, and instead are consistent with the suggestion that conserved parasite products such as haemozoin or GPI-anchors are the parasite-derived stimuli for cytokine induction.

## Introduction

Of the estimated 515 million clinical falciparum malaria infections each year, approximately 1 million cases are fatal (1). The reasons why some *Plasmodium falciparum* infections lead to death whereas most do not are not clearly understood (2). Increasingly, it is accepted that cytokines form part of the protective immune response directed against *P. falciparum* but that dysregulation of cytokine production may contribute to disease pathology (3). It has been shown that people differ in their propensity to produce cytokines in response to *P. falciparum* and that differential cytokine production can be associated with malaria disease manifestation (4). However, little is known about the contribution, if any, of parasite-dependent variation on induction of cytokines from human peripheral blood mononuclear cells (PBMCs).

Cytokines are thought to play an important role in the immune response to *P. falciparum*. Several *in vitro* studies show that production of pro-inflammatory cytokines (IFN-γ, IL-12 and TNF-α) after leukocyte stimulation with *P. falciparum* antigens is associated with reduced risk of clinical malaria infections (5,6) and high-density infections (6), as well as increased time to reinfection (7). Similarly, after experimental sporozoite infection of naïve volunteers, high peak serum levels of IFN-γ, TNF-α and IL-12 correlated with delayed time to parasite detection (4) suggesting that these cytokines contribute to the control of parasite growth. However, in the same study, fever was predominantly reported from people with high IFN-γ, and high ratios of IL-12 or TNF-α to IL-10 correlated with more rapid onset of clinical symptoms (4), suggesting that high or unregulated levels of pro-inflammatory cytokines can contribute to disease symptoms. Furthermore, studies of African patients reveal significantly different serum or plasma levels of the cytokines TNF-α, IL-12, IL-6 and IL-10 between children with severe malaria vs. children with uncomplicated malaria (8–10) suggesting that cytokine production may dictate both symptoms and manifestation of malaria infection. Interestingly, a higher ratio of IL-6 to IL-10 was also observed in sera of Vietnamese patients with fatal severe malaria, vs. those with severe disease who survived (11), implicating unbalanced pro-inflammatory and anti-inflammatory cytokine production as a risk factor for fatal malaria.

Regarding the source of variation in cytokine production in patients with malaria, it is known to be at least partly attributable to the propensity of the host to produce cytokines in response to *P. falciparum*. For example, several studies have shown that after *in vitro* stimulation of naïve donor PBMCs with *P. falciparum*, there is considerable variation between donors in both the amount of IFN-γ produced (12–14) as well as the proportion of IFN-γ responsive NK cells (12–15) and T cells (14). It has also been shown that for a given donor, the proportion of IFN-γ-producing NK cells after *P. falciparum* stimulation is consistent over time (15). Furthermore, variation in both total IFN-γ production and the proportions of IFN-γ-producing cell types after *P. falciparum*-induced PBMC stimulation has been observed in a semi-immune Papua New Guinean population (6). After experimental sporozoite infection of naïve volunteers, donor responses could be divided equally into those who produced moderate levels of IFN-γ and IL-10 with no IL-12p70, those who produced detectable IL-12p70 and high levels of IFN-γ and IL-10 and those who produced high levels of TGFβ in the absence of a pro-inflammatory response (4), providing further evidence for the existence of donor-dependent malaria-specific cytokine responses.

In rodent models of malaria, there is evidence that parasite strains vary in their ability to induce cytokines and that this variation affects disease manifestation and virulence. A nonlethal *P. yoelii* clone induces an initial pro-inflammatory response with later down-regulation by TGFβ, whereas a lethal *P. yoelii* clone in the same mouse strain is associated with high levels of TGFβ within 24 h and a reduced pro-inflammatory response (16). Similarly, induction of IFN-γ as well as the ratio of IFN-γ to IL-10 following *P. chabaudi* infection of mice is dependent on parasite clone, and differential cytokine induction partially explains the differences in virulence (weight loss) between clones (17).

For human malaria parasites, there is little existing evidence examining variation between parasite strains in cytokine induction. One early report showed that naïve donor TNF-α responses to *P. falciparum* field isolates can differ by over 100 fold after incubation with parasite lysate and suggested that parasites capable of inducing high levels of TNF-α were more likely to have come from patients with cerebral vs. uncomplicated malaria (18). During preliminary experiments to study the effect of the *P. falciparum* virulence-associated rosetting phenotype (19) on cytokine induction, we noted that some common laboratory strains varied in the magnitude of the IFN-γ response generated after co-culture with PBMCs, irrespective of the rosetting phenotype. We therefore set out to investigate the hypothesis that *P. falciparum* strains vary in their ability to induce cytokine responses from human PBMCs.

## Materials and methods

### *P. falciparum* strains

Nonrosetting *P. falciparum* parasites of strains TM284 (20), R29 (21), HB3 (http://www.wehi.edu.au/other_domains/MalDB/genomeInfo/FTPaccess/htmledFTP/Strains/pforigin.html) and Palo Alto (20) were used, derived by gelatin flotation or percoll purification of mixed rosetting and nonrosetting cultures and maintaining in culture the top layer of infected erythrocytes (22). These parasite strains each consist of a single genotype, and they are genetically distinct, except for R29 and Palo Alto, which are clones derived from the IT/FCR3 lineage that express different variant surface antigens (JA Rowe, unpublished observations). Palo Alto was originally derived from an African isolate; however, most of the ‘Palo Alto’ parasites now in general use (including those used here) were contaminated and overgrown by IT/FCR3 genotype parasites at some time in the past (23,24). Five field isolates frozen within 4 h of venepuncture as part of previous studies in east (19,25) and west (26) Africa were also used.

### *P. falciparum* culture

Laboratory strains were cultured in group O+ erythrocytes (Scottish National Blood Transfusion Service, Edinburgh, Scotland) and RPMI 1640 (Invitrogen, Paisley, UK) supplemented with 20 mm glucose (Sigma, Poole, UK), 2 mm glutamine (Invitrogen), 25 mm HEPES (Lonza, Basel, Switzerland), 25 μg/mL gentamycin (Lonza) with 10% pooled normal human serum (Scottish National Blood Transfusion Service) adjusted to pH 7·2–7·4 with NaOH (Sigma). Flasks were gassed with 1% O_2_/3% CO_2_/96% N_2_ and incubated at 37°C. Parasite maturity was assessed by examination of Giemsa-stained thin blood smears, and synchronicity was maintained by regular sorbitol lysis (27). Cultures were screened weekly for mycoplasma contamination (28) by PCR (Minerva Biolabs, Berlin, Germany) and were also tested for mycoplasma on the day of each experiment, and only mycoplasma-free cultures were used. For use in co-culture assays, parasite cultures at pigmented trophozoite stage and 10–20% parasitaemia were washed 3 times with warm RPMI 1640 supplemented with 20 mm glucose, 2 mm glutamine, 25 mm HEPES and 100 unit/mL penicillin/100 μg/mL streptomycin (Sigma) adjusted to pH 7·2–7·4 with NaOH and diluted with fresh erythrocytes to create either a range of parasitaemia (0·1–20%) or a specific parasitaemia (4% or 0·5%), determined by counts of at least 1000 cells on Giemsa-stained thin blood smears. Field isolates were thawed by standard methods (29) and cultured for one cycle *in vitro* to the pigmented trophozoite stage in supplemented RPMI 1640 with 10% pooled AB serum and diluted to 5% parasitaemia or 0·5% parasitaemia using group O+ erythrocytes prior to incubation with PBMCs as described below.

### PBMC preparation

Fifteen adult, malaria-naïve blood donors of European descent were recruited from the University of Edinburgh, having given written informed consent. Venous blood was collected into sterile heparinised tubes (Becton Dickinson, Oxford, UK), and PBMCs were separated by density centrifugation using Histopaque 1077. PBMCs were resuspended at 1 × 10^6^ cells/mL in RPMI 1640 with 20 mm glucose, 2 mm glutamine, 25 mm HEPES, 100 unit/mL penicillin/100 μg/mL streptomycin with 10% pooled normal human AB serum.

### 24 h co-culture assays over a range of parasitaemias

PBMCs were aliquoted at 5 × 10^5^ cells/well into flat-bottomed 48-well tissue culture plates (Greiner, Gloucestershire, UK) and incubated with 2·5 × 10^7^ erythrocytes infected with each of four strains of *P. falciparum* (TM284, R29, HB3, Palo Alto) at pigmented trophozoite stage over a range of parasitaemias (0·1–20%) for 24 h at 37°C, with an atmosphere of 5% CO_2_. Two or three replicate wells were set up for each parasitaemia and each parasite strain. Eleven donors were tested in five separate experiments. In all co-culture assays, 5 μg/mL phytohaemagglutinin (PHA; Sigma) and 2·5 × 10^7^ uninfected erythrocytes were included as positive and negative controls, respectively.

### Co-culture time courses at a single parasitaemia

To control for the effect of differences between parasite strains in the time taken to reach schizont rupture, co-culture assays were set up with multiple parasite strains per donor at a single parasitaemia (approximately 4% in two experiments and 0·5% in one experiment) and followed for 48 h, with time points every 6–8 h. Two or three wells were set up for each time point with each parasite strain. At each time point, supernatants were taken for IFN-γ ELISA, and parasite maturity was assessed from a Giemsa-stained thin blood smear that was examined by light microscopy. IFN-γ levels were measured in supernatants taken throughout the time course, with the levels at approximately 24 h after the start of schizont rupture for each strain being compared to examine strain variation.

### Cytokine ELISA

Initially, supernatant IFN-γ concentration was determined by ELISA using a BD OptEIA™ Human IFN-γ ELISA Set (BD Biosciences, Oxford, UK), according to the manufacturer’s instructions. Samples which exceeded the detection limit of the assay were diluted in 10% FCS in PBS (pH 7·0) and re-analysed. IFN-γ concentrations were calculated using the mean optical density of two wells and comparison with a standard curve.

### Multiplex analysis of cytokine concentration

Concentrations of IFN-γ, TNF-α, GM-CSF, IL-1β, IL-2, IL-4, IL-5, IL-6, IL-8, IL-10 and IL-17 were determined using multiplex luminescent bead kits [Human Cytokine 10-Plex supplemented with IL-17 detection reagents (Invitrogen)] according to the manufacturer’s instructions and analysed with a Luminex 200™ plate reader (Luminex, Austin, TX, USA). Fluorescence intensity was converted into cytokine concentration using xPONENT 3·1. The concentration of spontaneously bioactive TFGβ was determined using the same technique [Multi Species TGFβ1 Immunoassay Kit (Invitrogen)] whereas total TGFβ concentration was assayed following acidification and neutralization of samples, in accordance with manufacturer’s instructions, to dissociate TFGβ from its latent associated peptide.

### Transwell experiments

To determine the solubility of parasite antigens responsible for cytokine induction, PBMCs were physically separated from parasite cultures using a 0·4-μm Transwell membrane (Corning, Lowell, MA, USA). Soluble mediators can pass through the membrane, whereas larger products such as haemozoin and fragmented membrane cannot. PBMCs were aliquoted at 1 × 10^6^ cells/well into the bottom chamber, with 5 × 10^7^ erythrocytes infected with *P. falciparum* strain R29 (parasitaemia range 1–15%) in the top chamber. For comparison, equivalent numbers of PBMCs and infected erythrocytes were set up in wells without the Transwell membrane. After 24 h, IFN-γ production was measured by ELISA as described above, and the amount of cytokine produced in the presence of Transwells was compared to that from PBMC-parasite co-cultures with no Transwell divide.

### Statistical analysis

Statistical analysis of data was performed using Minitab 15 (Minitab Inc., State College, PA, USA). Analysis of the effect of PBMC donor, *P. falciparum* strain and parasitaemia on cytokine production was carried out using a general linear model (GLM), with mean cytokine concentration as the response variable, PBMC donor and parasite strain as main factors and parasitaemia as a covariate. To comply with the assumptions of this model, IFN-γ concentrations determined by ELISA were first normalized using Box–Cox transformation, whereas cytokine concentrations determined by multiplexed bead assay were subjected to log_10_ transformation. Heterogeneity and normal distribution of residuals were confirmed using Levene’s test and Kolmogrov–Smirnoff or Anderson-Darling tests, respectively. *Post hoc* pair-wise comparisons were performed using Tukey’s test. Correlations between cytokine levels were determined by Pearson correlation after normalization of data by log_10_ transformation. Additional parametric (anova) and nonparametric tests (Spearman rank correlation and Mann–Whitney *U* test) were used where indicated. To determine whether parasite strain and PBMC donor contributed to the variance in IFN-γ concentration measured at 24 h after the start of schizont rupture, data were examined using a GLM, with mean IFN-γ concentration (log_10_ transformation) as the response variable, and PBMC donor and parasite strain as main factors. Heterogeneity and normal distribution of residuals were confirmed as above. Graphical presentation of data was produced using graphpad prism 4 (GraphPad Software, La Jolla, CA, USA).

## Results

### Effect of parasite strain, parasitaemia and PBMC donor on the early IFN-γ response to *P. falciparum*

**Figure 1:**
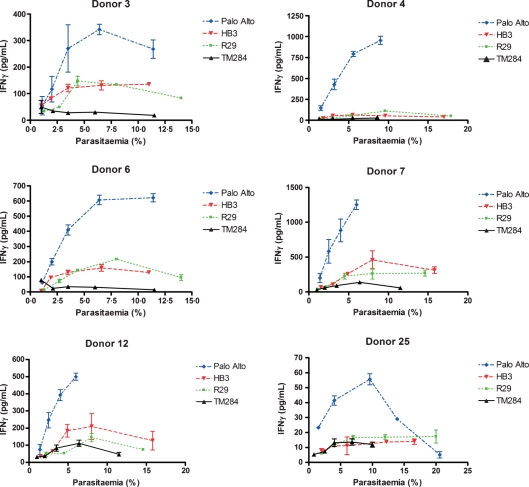
Production of IFN-γ after 24 h co-culture of human PBMCs with *P. falciparum*-infected erythrocytes is dependent on parasite strain. For 24 h, 5 × 10^5^ human PBMCs were stimulated with 2·5 × 10^7^ erythrocytes infected with one of four laboratory strains of *P. falciparum* (TM284, R29, HB3, Palo Alto) across a range of parasitaemias. The IFN-γ level in the culture supernatants was measured by ELISA. Across 11 donors (see Figure S1 for data from five additional donors), the IFN-γ response after PBMC stimulation with *P. falciparum* is significantly dependent on parasite strain (*F*_3,178_ = 48·49, *P* < 0·001). Error bars show the standard error about the mean of at least duplicate wells for each data point.

To determine whether parasite strain influences the magnitude of IFN-γ produced by PBMCs after *in vitro* co-culture with *P. falciparum*-infected erythrocytes, PBMCs from an individual donor were incubated for 24 h with four laboratory strains of *P. falciparum* (Palo Alto, HB3, R29 and TM284). The assays for all four strains were set up simultaneously (one strain per plate) over a range of parasitaemias (0·1–20%), with parasites at the mature pigmented trophozoite stage. For all 11 PBMC donors tested, there were consistent differences in the amount of IFN-γ elicited by the various strains. The data for six donors with the most closely matched range of parasitaemia across strains are shown in Figure 1, and the remaining five donors are shown in Figure S1. For all donors, the IFN-γ levels were highest after co-culture with strain Palo Alto, and lowest after co-culture with strain TM284 (Figures 1 and S1). Intermediate levels of cytokine were induced after co-culture with strains R29 and HB3 (Figures 1 and S1). Of note, levels of IFN-γ produced in response to strain TM284 were often barely detectable, even at the highest parasitaemia. For the other three parasite strains, a dose-dependent effect in relation to parasitaemia was seen, although for most donors, the amount of IFN-γ levelled off or reduced at the highest parasitaemia tested (for example Figure 1 donors 3, 6, 25), and these cases showed signs of nutrient depletion in the co-culture wells at the highest parasitaemia (yellow medium after 24 h). As expected from previous work (12–15), individual donors differed in the amount of cytokine produced, as shown by the differing *y* axis values in Figures 1 and S1.

General linear model (GLM) regression analysis (response variable: mean IFN-γ concentration; main factors: PBMC donor, parasite strain; covariate: parasitaemia) revealed that parasite strain significantly contributes to the variance in IFN-γ concentration (*F*_3,178_ = 48·49, *P* < 0·001). *Post hoc* pair-wise analysis showed that the IFN-γ response induced is significantly different between all strains (*P* < 0·001, Tukey’s test), except for strains HB3 and R29 (*P* = 1·0, Tukey’s test). Furthermore, regression analysis revealed that IFN-γ concentration is also significantly determined by PBMC donor (*F*_10,178_ = 28·06, *P* < 0·001) and the parasitaemia of the stimulating *P. falciparum* culture (*F*_1,178_ = 8·21, *P* = 0·005). The larger *F* statistic associated with parasite strain indicates that parasite strain contributes to more of the variance in IFN-γ concentration than donor, with parasitaemia making a lesser contribution than the other two factors.

### Effect of parasite strain, parasitaemia and PBMC donor on production of other cytokines

Having shown that the production of the pro-inflammatory cytokine IFN-γ is dependent on *P. falciparum* strain, we hypothesized that production of other cytokines may also be influenced in a strain-specific manner. We therefore used a multiplex cytometric bead assay to determine the concentrations of various cytokines (IFN-γ, TNF-α, GM-CSF, IL-1β, IL-2, IL-4, IL-5, IL-6, IL-8, IL-10 and IL-17) in the PBMC: infected erythrocyte co-culture supernatants described above. Sufficient material was available to test eight of the above 11 donors.

For all eight donors with all parasite strains, very little IL-2, IL-4, IL-5 and IL-17 could be detected and therefore these cytokines were not included in subsequent analysis. Levels of IL-8 exceeded the detection range of the assay and technical reasons prevented further analysis of this cytokine. Detectable levels of IFN-γ, IL-1β, IL-6, GM-CSF, TNF-α and IL-10 were found in supernatants from all donors. The levels of IFN-γ measured by ELISA and multiplex bead assay were compared to determine whether the assays showed comparable sensitivity. IFN-γ concentrations measured by ELISA and multiplex bead assays were significantly positively correlated (ρ = 0·964, *P* < 0·0001, Spearman rank correlation), although in general, the multiplex assay gave values approximately 10-fold lower than the ELISA. The multiplex values of IFN-γ are used in the following analyses in relation to the other cytokines measured by the multiplex assay.

**Figure 2:**
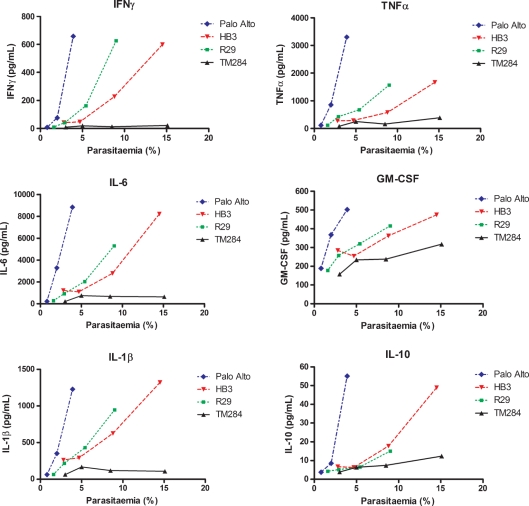
Other cytokines show strain-dependent patterns similar to IFN-γ after 24 h co-culture of human PBMCs with *P. falciparum-*infected erythrocytes. For 24 h, 5 × 10^5^ human PBMCs were stimulated with 2·5 × 10^7^ erythrocytes infected with one of four laboratory strains of *P. falciparum* (TM284, R29, HB3, Palo Alto) across a range of parasitaemias. IFN-γ, TNF-α, IL-6, GM-CSF, IL-1β and IL-10 levels in the culture supernatants from eight donors were measured by multiplex bead assay. Data from one donor (donor 9) are shown, and the remaining donors are shown in Figure S2. Across all donors, the cytokine response after PBMC stimulation with *P. falciparum* is significantly dependent on parasite strain (*P* < 0·001 for each cytokine, see Table S1).

The absolute levels of cytokines elicited in response to *P. falciparum* varied depending on the cytokine (see the *y* axis in Figure 2); however, overall, very similar patterns of strain variation were seen across all cytokines (Figure 2, data from donor 9 and Figure S2, data from other seven donors). As with IFN-γ, Palo Alto induced the highest level of cytokines, TM284 the least, and HB3 and R29 were intermediate. When IFN-γ levels were correlated with the other cytokines, significant positive correlations were seen in all cases (IL-1βρ = 0·738, *P* < 0·001; IL-6 ρ = 0·687, *P* < 0·001; GM-CSF ρ = 0·675, *P* < 0·001 and TNF-αρ = 0·834, *P* < 0·001) as determined by Pearson correlation after log_10_ normalization of all cytokine data. Furthermore, levels of pro-inflammatory cytokines significantly positively correlated with each other (GM-CSF vs. IL-1βρ = 0·890, *P* < 0·001; GM-CSF vs. IL-6 ρ = 0·898, *P* < 0·001; GM-CSF vs. TNF-αρ = 0·860, *P* < 0·001; IL-1β vs. IL-6 ρ = 0·939, *P* < 0·001; IL-1β vs. TNF-αρ = 0·992, *P* < 0·001; IL-6 vs. TNF-αρ = 0·920, *P* < 0·001 Pearson correlation). Similar to the data for IFN-γ, and as expected given the high level of correlation between the pro-inflammatory cytokines, multiple regression analysis using a GLM revealed that after 24 h of PBMC co-culture with *P. falciparum*, the variance in IL-1β, IL-6, GM-CSF and TNF-α levels was significantly determined by parasite strain, PBMC donor and parasitaemia (Table S1).

We also investigated the production of the anti-inflammatory cytokines IL-10 and TGFβ to determine whether raised levels of anti-inflammatory cytokines might explain the limited pro-inflammatory response to parasite strain TM284 seen in Figures 1 and 2. IL-10 was measured as part of the multiplex bead assay described above. IL-10 levels were found to be significantly lower than IFN-γ levels (IL-10 range 2–150 pg/mL, IFN-γ range 2–650 pg/mL, *P* = 0·023 Wilcoxon signed rank test). However, IL-10 levels were significantly positively correlated with the levels of pro-inflammatory cytokines (IL-10 vs. IFN-γρ = 0·653, *P* < 0·001; IL-10 vs. GM-CSF ρ = 0·694, *P* < 0·001; IL-10 vs. IL-1βρ = 0·762, *P* < 0·001; IL-10 vs. TNF-αρ = 0·740, *P* < 0·001; IL-10 vs. IL-6 ρ = 0·712, *P* < 0·001 Pearson correlation), across all strains, including TM284. In other words, the low pro-inflammatory responses to TM284 could not be explained by high levels of IL-10 (see Figure 2 for an example from donor 9, showing that the patterns of IL-10 induction by the four parasite strains mirror the patterns for the pro-inflammatory cytokines. Seven additional donors are shown in Figure S2). Assays were also carried out to detect bioactive and total TGFβ; however, bioactive TGFβ was not detectable, and the variance in total TGFβ was not significantly influenced by parasite strain (*F*_3,84_ = 1·24, *P* = 0·3) or parasitaemia (*F*_1,84_ = 0·38, *P* = 0·539) (Figure S3), although it was donor dependent (*F*_5,84_ = 11·18, *P* < 0·0001).

### The effect of schizont rupture on cytokine production

**Figure 3:**
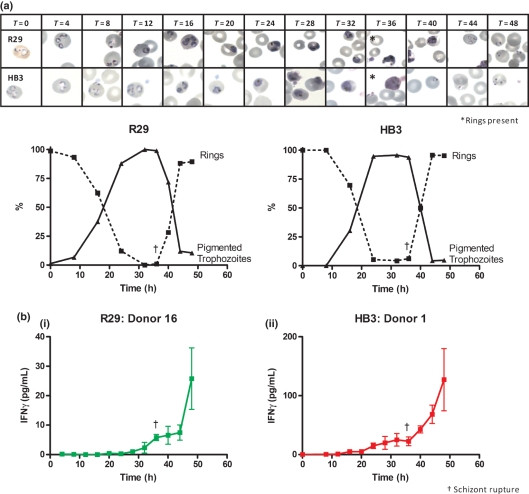
Time course of IFN-γ production by human PBMCs in relation to *P. falciparum* maturity. (a) 2.5 × 10^7^ erythrocytes infected with strain R29 or HB3 at 4% parasitaemia were incubated with 5x10^5^ human PBMCs from a single donor. Time points were taken 4-8 hourly over a 48 hour period, and parasite maturity at each time point was assessed by examining a Giemsa stained thin film by light microscopy. Representative images for each parasite strain and each time point are shown in the upper panel. The percentage of rings and pigmented trophozoites/schizonts for each parasite strain at each time point were counted from the Giemsa stained thin film and are shown in the lower panel. (b) IFN-γ level in culture supernatants at each time point was measured by ELISA. Error bars show the standard error about the mean of at least duplicate wells for each data point. For both strains, HB3 and R29 schizont rupture began at 36 h (indicated with †) and IFN-γ production increased substantially after 40 h. Similar data were obtained from additional PBMC donors with each of these *P. falciparum* strains (not shown).

Having found a potentially strain-specific component responsible for cytokine induction, attention was directed at identifying the stimulus for cytokine induction. To investigate which component of the parasite life cycle is responsible for the stimulation of cytokine production by PBMCs, time courses examining cytokine production in relation to parasite maturity were carried out. PBMCs were co-incubated with ring stage parasites at 4% parasitaemia for 48 h, enabling parasites to mature through pigmented trophozoite stage, schizogeny and reinvasion (Figure 3a). Using two strains of *P. falciparum* (HB3 and R29), it was found that although low levels of IFN-γ production could be detected prior to schizogony, cytokine production was greatly enhanced after schizont rupture, which began at about 36 h for both parasite strains in this experiment (Figure 3b). For strain R29, IFN-γ production in the 12 h prior to schizont rupture (median, 0·7 pg/mL; range 0·43–2·33 pg/mL) was significantly lower than that produced in the 12 h after schizont rupture (median, 7·02 pg/mL; range 5·8–25·8 pg/mL; Mann–Whitney *U* test, *P* = 0·030). Similarly for strain HB3, IFN-γ levels were lower in the 12 h prior to schizont rupture (median, 17·43 pg/mL; range 5·07–25·02 pg/mL) than the 12 h post-schizont rupture (median, 55·58 pg/mL; range 22·58–127·07 pg/mL; Mann–Whitney *U* test, *P* = 0·061). Of note, it can be seen that schizont rupture precedes the sharp increase in cytokine production by up to 8 h (Figure 3). This time lag of 8–12 h before cytokine levels increase is also seen when PBMCs are stimulated with the mitogen PHA (data not shown).

### Investigation of the effect of the timing of schizont rupture on parasite strain variation in cytokine induction

Having demonstrated that cytokine production is increased after schizont rupture and that a time lag exists between schizont rupture and cytokine upregulation, we considered the possibility that the strain-dependent differences in cytokine production noted above could result from differences in the time taken to reach schizont rupture between the parasite strains tested. This was a particular concern because during long-term cultures of the high IFN-γ-inducing Palo Alto strain, we had regularly observed that this parasite strain matures rapidly and has a very short blood stage life cycle, in the range of 35–40 h, rather than the 48 h thought to be normal for *P. falciparum*.

**Figure 4:**
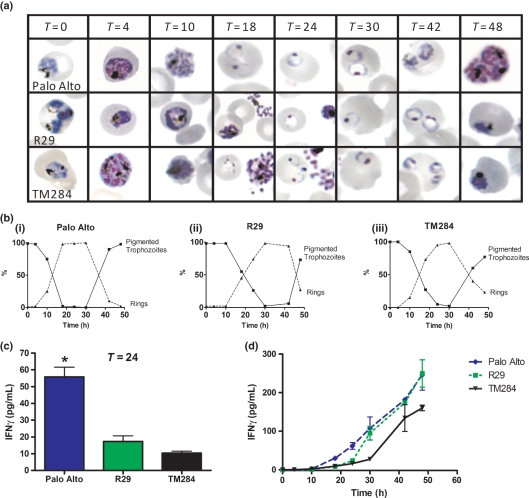
Time course of IFN-γ production by human PBMCs in response to *P. falciparum* strains Palo Alto, R29 and TM284 over 48 h. (a) 2·5 × 10^7^ erythrocytes infected with strain Palo Alto, R29 or TM284 at 4% parasitaemia were incubated with 5 × 10^5^ human PBMCs from a single donor. Time points were taken 6–8 hourly, and parasite maturity at each time point was assessed by examining a Giemsa-stained thin film by light microscopy. Representative images for each parasite strain and each time point are shown. (b) The percentage of rings and pigmented trophozoites/schizonts for each parasite strain at each time point were counted from the Giemsa-stained thin film. Palo Alto completes schizogeny after 18 h, whereas R29 and TM284 complete schizogeny only after 30 h. (c and d) IFN-γ level in culture supernatants at each time point was measured by ELISA. Error bars show the standard error about the mean of at least duplicate wells for each data point. (c) At 24 h after the start of culture, which is the standard time point used in Figures 1 and 2, Palo Alto induced higher levels of IFN-γ than TM284 and R29 (*P = 0·007, anova). (d) After 48 h of co-culture, IFN-γ induction by strains R29 and Palo Alto is indistinguishable. IFN-γ produced in response to TM284 is comparable to that induced by Palo Alto and R29 although delayed by approximately 10 h.

To investigate the relationship between parasite maturation rate, *P. falciparum* strain and IFN-γ induction, a time course was set up incubating PBMCs from a single donor with infected erythrocytes of strains TM284, R29 and Palo Alto synchronized at pigmented trophozoite stage at 4% parasitaemia and of approximately equal maturity as determined by Giemsa smear (Figure 4a, *T* = 0). Close inspection of the rate of parasite maturation showed that strain Palo Alto had begun schizont rupture by 10 h, and completed schizogeny (i.e. was 100% ring stage parasites) by 18 h (Figure 4b). However, for both strains R29 and TM284, schizont rupture did not begin until 10–18 h, and schizogeny was not completed until about 30 h (Figure 4b). Therefore, despite apparent equivalent maturity of all strains at the start of the assay, strain Palo Alto reached schizont rupture more rapidly than the other two stains.

IFN-γ levels were measured in the culture supernatant at each time point for each parasite strain. After 24 h of co-culture, which is the standard time point used in the previous assays, higher levels of IFN-γ were induced by strain Palo Alto than by strains R29 and TM284 (Figure 4c, *P* = 0·007, anova), consistent with previous results. Across the whole time series, however, it can be seen that R29 induced equivalent levels of IFN-γ to Palo Alto by 30 h (Figure 4d) and that strain TM284 also induced IFN-γ, although its response was slightly delayed compared to R29 (Figure 4d). If the amount of IFN-γ produced by each strain was compared at approximately 24 h post-schizont rupture (rather than after 24 h of co-culture), Palo Alto (107 pg/mL at *T* = 30) did not show higher levels of IFN-γ compared to R29 (173 pg/mL *T* = 42) and TM284 (134 pg/mL *T* = 42) (*P* = 0·183 anova, these data are shown as experiment A in Table 1). These data suggest that the apparent greater capacity of Palo Alto to induce cytokines compared to the other strains (shown in Figures 1 and 2) may be because of its more rapid development to schizogony than the other strains, rather than any intrinsic difference in the type or amount of parasite antigen that stimulates the early cytokine response.

**Table 1:** Assessment of IFN-γ levels at approximately 24 h after the start of schizont rupture in three independent experiments using multiple parasite strains

Experiment^a^	Parasite Strain	Parasitaemia and maturity at start of experiment	Time point approx 24 h after schizont rupture	IFN-γ 1 pg/mL^b^	IFN-γ 2 pg/mL^b^	Mean IFN-γ pg/mL
A	Palo Alto	4·0% mature pigmented trophozoites	30 h	86·1	128·3	107·2
A	TM284	4·5% mature pigmented trophozoites	42 h	158·5	109·5	134·0
A	R29	4·0% mature pigmented trophozoites	42 h	171·9	174·4	173·2
B	Palo Alto	2·0% bursting schizonts, 22·0% small rings	18 h	1459·7	ND	1459·7
B	TM284	4·4% bursting schizonts, 3·8% small rings	18 h	3670·1	3370·1	3520·1
B	Field isolate A	5·8% schizonts	32 h	851·9	828·6	840·3
B	Field isolate B	5·1% schizonts	32 h	762·3	1015·6	889·0
B	Field isolate C	4·4% mature pigmented trophozoites	Mean of 32 and 40 h	939·6	801·3	870·5
C	Palo Alto	1·3% large rings	42 h	5·0	38·3	21·7
C	TM284	0·5% mature pigmented trophozoites	42 h^c^	5·2	2·5	3·9
C	Field isolate D	0·5% mature pigmented trophozoites	42 h^c^	70·9	58·4	64·6
C	Field isolate E	0·7% schizonts	24 h	65·0	58·2	61·6

a Each experiment used a different single donor. Experiment A is shown in detail in Figure 4. Experiments B and C are as described in the text;

b IFN-γ levels in culture supernatants of two replicate wells were measured at each time point by ELISA;

c For both TM284 and Field isolate D, the final time point studied (42 h) was about 18 h after schizont rupture began. Ideally, a 48-h time point (giving data 24 h after schizont rupture) would have been optimal; however, this was not available for this experiment.

It is apparent from these data that assays using a set time period after co-culture (such as carried out for Figures 1 and 2), without taking into account differences in parasite maturation rate, are problematic. It would be more appropriate (although more demanding technically) to compare cytokine levels at a standard time point after schizont rupture has begun. To investigate further whether strain variation in ability to induce cytokines occurs when timing of schizogony is controlled for, we set up two further experiments in which multiple strains were compared within a single experiment with a single donor, and parasite maturity was closely monitored over 48 h to allow assessment of IFN-γ at approximately 24 h after schizont rupture had begun. The first experiment (B in Table 1) compared Palo Alto, TM284 and three field isolates from west Africa, and the second experiment (C in Table 1) compared Palo Alto and TM284 with two field isolates from east Africa. These experiments are not ideal, because the parasitaemia of each strain was not perfectly matched, and the maturity of the parasite strains varied at the start of the experiment (Table 1). However, it is technically extremely difficult to obtain several parasite strains at equivalent levels of maturity, especially when using field isolates which are unpredictable in the time it takes them to mature to schizogony. Two of the field isolates used in these studies developed at very similar rates, and their data are shown over the whole time course in Figure S4. These two field isolates matured at equivalent rates and showed very similar levels of IFN-γ over the first 40 h of the time course (Figure S4). For all the parasite strains used in experiments B and C, the IFN-γ levels were measured 6–8 hourly over 48 h, and parasite maturity assessed from Giemsa-stained thin films at each time point. The approximate time point at which schizont rupture began was assessed from the Giemsa-stained thin films, and the IFN-γ levels approximately 24 h after that point are shown in Table 1. Given the difficulties in standardizing conditions, the results of these experiments must be considered preliminary. However, the data from the three experiments in Table 1 can be usefully examined using a GLM analysis (response variable: mean IFN-γ concentration at 24 h post-schizont rupture; main factors: PBMC donor and parasite strain). This analysis showed that when assessed at approximately 24 h after schizont rupture, parasite strain does not have a significant effect on the variance in IFN-γ levels (*F*_7,11_ = 3·66, *P* = 0·383), but PBMC donor remained significant (*F*_2,11_ = 23·44, *P* < 0·001). In particular, these experiments also show that despite the original findings in Figures 1 and 2, parasite strain TM284 is able to induce IFN-γ from PBMCs similar to the other strains when parasites of adequate maturity are examined (Experiments A and B in Table 1).

### Investigation of the nature of the *P. falciparum* antigen responsible for cytokine induction

**Figure 5:**
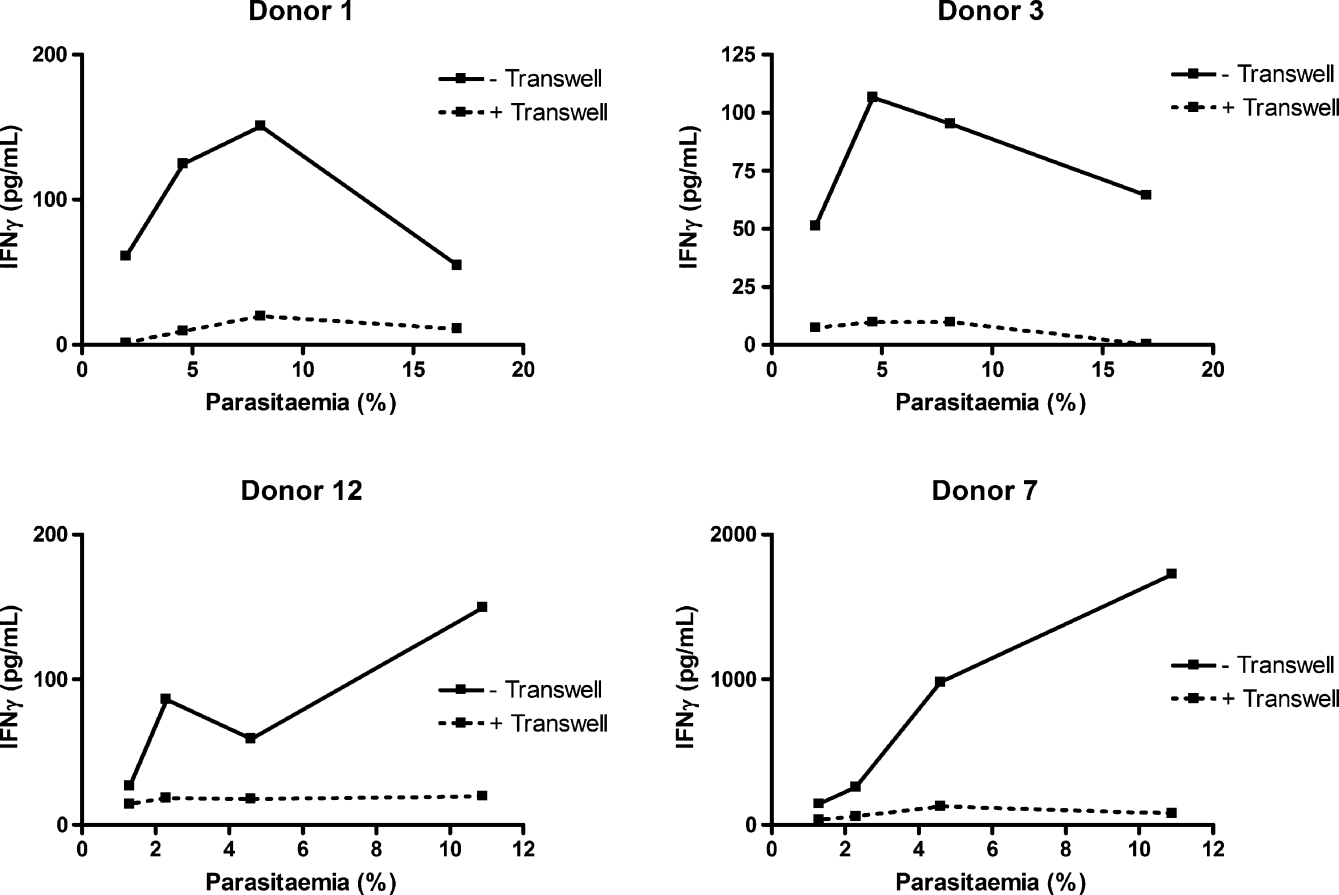
PBMC IFN-γ production in response to *P. falciparum*-infected erythrocytes is significantly reduced in the presence of a 0·4-μm Transwell membrane. For 24 h, 1 × 10^6^ human PBMCs were co-cultured with 5 × 10^7^ infected erythrocytes of *P. falciparum* strain R29 (parasitaemia range 1–17%) either with or without a Transwell. Supernatant IFN-γ concentrations were assessed by ELISA. Across four donors, IFN-γ levels were significantly lower in the presence of a Transwell membrane (Mann–Whitney *U* test, *P* < 0·001). +Transwell: PBMCs (bottom chamber) and infected erythrocytes (top chamber) were separated by a 0·4-μm Transwell membrane. −Transwell: No separation of PBMCs and infected erythrocytes.

Having shown that cytokine induction is at its highest level after schizont rupture (Figure 3b) and that parasite strains do not vary significantly in ability to induce cytokines if the timing of schizont rupture is controlled for (Figures 4 and S4 and Table 1), the nature of the parasite antigen responsible for cytokine induction was investigated. To assess whether the antigen was soluble, the cytokine response of PBMCs physically separated from infected erythrocytes using a 0·4-μm Transwell membrane was compared to that of PBMCs co-cultured in contact with infected erythrocytes. As a positive control, addition of soluble PHA (5 μg/mL) to the top chamber of the Transwell resulted in IFN-γ production from PBMCs that was indistinguishable from that induced by PHA in the absence of a Transwell divide (260 pg/mL without Transwell and 265 pg/mL with Transwell). This demonstrated that soluble mediators are able to pass through the Transwell membrane. In contrast, the IFN-γ response to *P. falciparum*-infected erythrocytes (strain R29) was significantly reduced when the PBMCs were physically separated from the infected erythrocytes by a Transwell (Figure 5, with Transwell, median IFN-γ 15·9 pg/mL; range 0–126·4 pg/mL) compared to co-culture with no Transwell (median IFN-γ 100·8 pg/mL; range 51·2–1726·2 pg/mL; Mann–Whitney *U* test, *P* ≤ 0·001). Further controls showed that infected erythrocytes and PBMCs co-cultured together in either the top or the bottom chambers gave equivalent responses to those seen when no Transwells were present (data not shown), indicating that the Transwell does not nonspecifically inhibit the PBMC cytokine response.

## Discussion

The aim of this study was to investigate the effect of parasite strain on the early innate cytokine response to *P. falciparum*. Several studies have noted that malaria-specific cytokine production varies between people; however, this is the first study to fully investigate the contribution of parasite strain-specific factors on induction of early cytokine responses. We found that after 24 h co-culture of PBMCs with infected erythrocytes of four *P. falciparum* laboratory strains, there was evidence for consistent strain variation in the amount of IFN-γ induced (Figures 1 and S1). PBMC donor and levels of parasitaemia of the stimulating culture also had significant effects on the amount of cytokine detected. In addition, we found that IFN-γ levels were highly positively correlated with the levels of other pro-inflammatory cytokines (IL-1β, IL-6, GM-CSF and TNF-α) and that parasite strain, PBMC donor and parasitaemia also had significant effects on the levels of the other cytokines (Figures 2 and S2 and Table S1). However, further experiments paying particular attention to parasite maturation rates showed that the highly inducing parasite strain (Palo Alto) reached schizont rupture more rapidly than the others strains (Figure 4). When IFN-γ levels were compared at 24 h after the start of schizont rupture (rather after 24 h of co-culture), significant strain effects were no longer detected (Table 1). The poorly inducing parasite strain TM284 was found to induce IFN-γ similar to other strains when sufficiently mature parasites were used. We therefore conclude that although strain variation in cytokine induction can be detected in 24 h co-culture assays, this difference in largely because of variation between strains in the time it takes the parasites to reach schizont rupture, rather than any intrinsic difference between strains in the antigens that induce cytokine production.

Many previous studies on early innate immune responses to *P. falciparum*-infected erythrocytes have used schizont-stage parasites in co-culture assays (6,12–15), although some use mixed pigmented trophozoite and schizont-stage cultures (30,31). The precise stage of parasites used was not critical in the previous studies, because in most cases, only a single parasite strain was used and the aim was to examine aspects of the human immune response rather than parasite variation. However, for future work, the data shown here indicate that if the aim of the experiment is to compare parasite strains, then parasite maturity is of paramount importance. If we had set up experiments with all parasite strains at the schizont stage, then the misleading results of Figures 1 and 2 might have been avoided. However, it is technically demanding to synchronize multiple strains to exactly the same lifecycle stage, and even within ‘schizont stage’ parasites, variation in maturity or synchronicity of cultures might influence outcome (for example, a highly synchronized culture of bursting schizonts might give different results to an equivalent culture of highly synchronized early segmenting parasites or a culture with mixed early and bursting schizonts). It is possible that chemical agents such as aphidicolin (32) that reversibly arrest parasites at a fixed stage in development could be used to overcome some of the technical problems inherent in preparing multiple parasite strains at the same level of maturity.

It was notable in this study that the high cytokine-inducing strain Palo Alto consistently matured more rapidly and reached schizont rupture earlier than the other strains examined. We estimate that the asexual blood stage cycle duration for strain Palo Alto is approximately 35–40 h, rather than the 48 h considered normal for *P. falciparum*. Whether differences in blood stage asexual lifecycle length occur in natural parasite populations and affect parasite virulence and clinical disease manifestation is unknown, and would be difficult to test. Parasites require a period of adaptation to *in vitro* conditions before they can be successfully propagated in long-term cultures, and many fail to adapt. Variation in the asexual cycle length after adaptation to culture could be because of mutations acquired during the adaptation process, rather than because of naturally occurring intrinsic differences between parasite strains. It would be possible to investigate the asexual cycle length of fresh clinical isolates in their first cycle of *in vitro* growth; however, this could be affected by extraneous factors such as exposure to anti-malarial drugs before the parasite sample was collected from the patient. Previous work has shown that the multiplication rate of *P. falciparum* field isolates varies and that high multiplication rates are associated with more severe clinical symptoms in a low transmission area (33) but not in a moderate transmission area (29). However, to our knowledge, there are no data examining variation in cycle length and whether this influences parasite virulence and disease manifestation.

It was well documented during malaria therapy treatment of patients with neurosyphilis that different parasite strains consistently differed in the severity of clinical symptoms that they caused (34), providing evidence that *P. falciparum* strains differ in virulence. However, the precise mechanisms of parasite virulence remain unknown, although may relate to adhesion properties such as rosetting (19,35,36), variant surface antigen expression (37,38), immunomodulation of host cells (39) or parasite multiplication rates (29,33) and selectivity of invasion (40,41). Investigation of other potential virulence factors such as multiplicity of infection (42,43) or invasion phenotype profiles (41,44) has so far yielded negative results, while others such as platelet-mediated clumping remain controversial (45,46).

Despite much recent interest in the early cytokine response to *P. falciparum,* the precise stimulus for cytokine production remains unclear. Live schizont-stage parasites are better inducers of innate cell activation than dead schizonts or parasite lysates (12,47,48), suggesting that the process of schizont rupture or contact with whole infected cells is required for immune cell activation (13). Candidate cytokine inducers include glycophosphatidyl (GPI) anchors of proteins released at schizont rupture (49), haemozoin (50) and haemozoin-bound parasite DNA (51). In this study, we have shown that although cytokines can be detected prior to schizont rupture, cytokine production is significantly increased after schizogeny, consistent with the findings reported elsewhere (4,48,52). In addition, our data indicate that, after controlling for variation in the timing of schizont rupture, there is no evidence for strain variation in cytokine induction by *P. falciparum*. Furthermore, our data suggest that soluble parasite products released at schizont rupture are not responsible for most of the early cytokine response, because physical separation of PBMCs from infected erythrocytes by a Transwell inhibits IFN-γ induction (Figure 5). These findings are consistent with the parasite cytokine-inducing antigens being conserved, insoluble parasite products such as haemozoin or membrane fragments containing GPI anchors.

One previous article examined strain variation in cytokine induction and noted a clear difference in the ability to induce TNF-α between rosetting and nonrosetting subpopulations of the parasite clone R29, whereas this difference was not seen in a genetically distinct clone Malayan Camp (53). We later discovered that the R29 clone was infected with mycoplasma (28), therefore, it is possible that the difference in cytokine-inducing ability of the parasite subpopulations was because of higher mycoplasma load in the rosetting population (because the enrichment procedure for rosetting could also enrich for mycoplasma). Another article by the same authors showed that parasite lysates derived from field isolates in the first cycle of *in vitro* growth varied in the amount of TNF-α they induced from PBMCs by almost 100-fold (18). It seems unlikely that mycoplasma was the source of the variation in this case (because they are usually contaminants of long-term cell lines rather than short-term cultures); however, its is possible that differences in the timing of schizont rupture between isolates could explain at least some of the variation in cytokine induction. Taking these data, together with our current study, we conclude that there is currently no strong evidence to support strain variation in cytokine induction by *P. falciparum*. However, we cannot exclude the possibility that a more widespread survey of parasite strains, which excluded mycoplasma contamination and controlled for timing of schizont rupture, might uncover some strain variation in innate immune cell activation. Such an experiment using live, whole infected erythrocytes would be technically challenging to undertake.

The finding in this study that PBMC donor contributes to the variance in the concentration of IFN-γ produced by PBMCs after *P*. *falciparum* co-culture supports previous reports that the cytokine production in response to *P. falciparum* varies in different people (12–15). As most cytokine production within the first 24 h of PBMC stimulation with malaria parasites is derived from components of the innate immune response NK cells (12) and γδT cells (14,48) this is perhaps indicative of differential expression of the receptor for *P. falciparum* components (currently unidentified) on these cells. Alternatively, these data could be explained by differential macrophage activation between donors, leading to variation in bystander activation of innate cells by macrophage-derived cytokines (54).

This study also found that the amount of cytokines produced by PBMCs after 24 h of PBMC-parasite co-culture is dependent on the parasitaemia of the starting culture. Earlier studies have used purified infected erythrocyte preparations (i.e. 100% parasitaemia) to stimulate cytokine induction (12,13), and it was suggested that the presence of uninfected erythrocytes profoundly inhibits the ability of *P. falciparum* to induce innate immune responses (55). Despite the presence of uninfected erythrocytes in our assays, we were able to detect cytokine induction over a wide range of parasitaemias (0·1–20%). For most donor responses shown here, cytokine responses increased with levels of parasitaemia until a parasitaemia of approximately 7% after which levels of cytokine production declined. This is consistent with a previous study that found that a ratio of three infected erythrocytes to one PBMC was optimal for maximum IFN-γ detection (12). This ratio represents an equivalent number of infected erythrocytes as that present at a parasitaemia of approximately 6% in our system. The increasing levels of IFN-γ seen with increasing parasitaemia up to 7% are likely to be because of higher numbers of parasites and parasite-derived stimuli. At high parasitaemias, it is likely that the parasites and PBMCs compete for resources, with a detrimental effect on PBMC viability (we observed yellow medium at the end of 24 h in wells with a high parasitaemia). More importantly, however, the finding that level of parasitaemia is a determinant of levels of cytokine production may have wider implications for field studies. It seems intuitively obvious that more parasites will mean more cytokines; however, surprisingly, some previous reports on cytokine levels in relation to malaria severity have not included a detailed analysis of the effect of different levels of parasitaemia between patients (10,56). Interestingly, although it is apparent that patients from malaria-endemic sub-Saharan African countries can experience very high parasitaemia without severe disease symptoms and associated mortality (57,58), this is not the case for patients from low malaria transmission areas or nonimmune travellers where increasing parasitaemia is predictive of increasing disease severity and death (59,60). The finding that plasma levels of TNF-α and IL-10 but not IL-12 positively correlate with parasitaemia (9) as well as contradictory findings relating nonsevere hyperparasitaemia to both low (10) and high (11) levels of IL-6 suggests that in future studies, more attention should be paid to the effect of levels of parasitaemia on the levels of serum or plasma cytokines measured.

After 24 h of parasite-PBMC co-incubation, this study found the levels of pro-inflammatory cytokines (IFN-γ, TNF-α, IL-6, IL-1β and GMC-SF) produced correlated with each other, as well as with IL-10. This is in support of an additional study which noted that after *P.* *falciparum* stimulation of PBMCs from Ghanaian donors, levels of TNF-α, IFN-γ and IL-12 were strongly correlated within donors (5) and is suggestive that production of pro-inflammatory cytokines is either co-regulated or forms part of an inflammatory cascade. However, the finding of the study presented here that levels of IL-10 are positively correlated with levels of pro-inflammatory cytokines differs from previous work (5). This discrepancy might be related to use of PBMC donors with different levels of malaria exposure in the two studies, which could be investigated further.

In conclusion, this study found no firm evidence for the existence of strain variation in the ability of *P. falciparum*-infected erythrocytes to induce cytokines in short-term co-cultures with PBMCs and highlights the importance of considering parasite maturity and lifecycle length when assessing and comparing cytokine responses between different parasite strains.

## SUPPORTING INFORMATION

**Figure S1.** IFN-γ levels after 24 h co-culture of *P. falciparum* infected erythrocytes with human PBMCs for five additional donors. 5 × 10^5^ human PBMCs were co-cultured with 2.5 × 10^7^ erythrocytes infected with one of four laboratory strains of *P. falciparum* (TM284, R29, HB3, Palo Alto) across a range of parasitaemia for 24 h. The IFN-γ level in the culture supernatants were measured by ELISA. Across all 11 donors (the other six donors are shown in Figure 1) the IFN-γ response after PBMC stimulation with *P. falciparum* was significantly dependent on parasite strain (*F*_3,178_ = 48.49, *P* < 0.001). Error bars (where shown) show the standard error about the mean of at least duplicate wells for each data point.

**Figure S2.** Cytokine production after 24 h co-culture of *P. falciparum* infected erythrocytes with human PBMCs for seven additional donors. 5 × 10^5^ human PBMCs were stimulated with 2.5 × 10^7^ erythrocytes infected with one of four laboratory strains of *P. falciparum* (TM284, R29, HB3, Palo Alto) across a range of parasitaemia for 24 h. IFN-γ, TNF-α, IL-6, GM-CSF, IL-1β and IL-10 levels in the culture supernatants from eight donors were measured by multiplex bead assay. Data from seven donors are shown (see Figure 2 for data from an additional donor). Across all donors, the cytokine response after PBMC stimulation with *P. falciparum* is significantly dependent on parasite strain (*P* < 0.001 for each cytokine, see Table S1).

**Figure S3.** Total TGFb levels after 24 h co-culture of *P. falciparum* infected erythrocytes with human PBMCs. 5 × 10^5^ human PBMCs were co-cultured with 2.5 × 10^7^ erythrocytes infected with one of four laboratory strains of *P. falciparum* (TM284, R29, HB3, Palo Alto) across a range of parasitaemia for 24 h. Total TGFb levels in the culture supernatants were measured by multiplex bead assay. The results shown are from donor 3, and the five other donors tested showed similar patterns. Across all six donors the variance in TGFb levels was not significantly affected by parasite strain (*F*_3,84_ = 1.24, *P* = 0.3) or parasitaemia (*F*_1,84_ = 0.38, *P* = 0.539), but was significantly affected by PBMC donor (*F*_5,84_ = 11.18, *P* < 0.001).

**Figure S4.** IFN-γ produced by human PBMCs after 46 h co-culture with two *P. falciparum* field isolates from African patients. 2.5 × 10^7^ erythrocytes infected with either field isolate A or B at 5% parasitaemia were co-cultured with 5 × 10^5^ human PBMCs from a single donor over 46 h. (a) Parasite maturity was assessed every 6–8 h throughout the time course from Giemsa stained thin films. The parasitaemia and percentage rings and pigmented trophozoites/schizonts were counted at each time point. (b) The IFN-γ level in culture supernatants was measured by ELISA at each time point. Error bars show the standard error from duplicate wells.

**Table S1.** Output of GLM regression analysis assessing the contributions of PBMC donor, parasite strain and parasitaemia on production of pro-inflammatory cytokines after 24 h co-culture of *P. falciparum* with human PBMCs.
