# Supplementary figures and images for: Strain variation in early innate cytokine induction by Plasmodium falciparum
Source: Parasite Immunol. 2010 Jul;32(7):512–27. doi: 10.1111/j.1365-3024.2010.01225.x (PMC2941733; doi:10.1111/j.1365-3024.2010.01225.x)

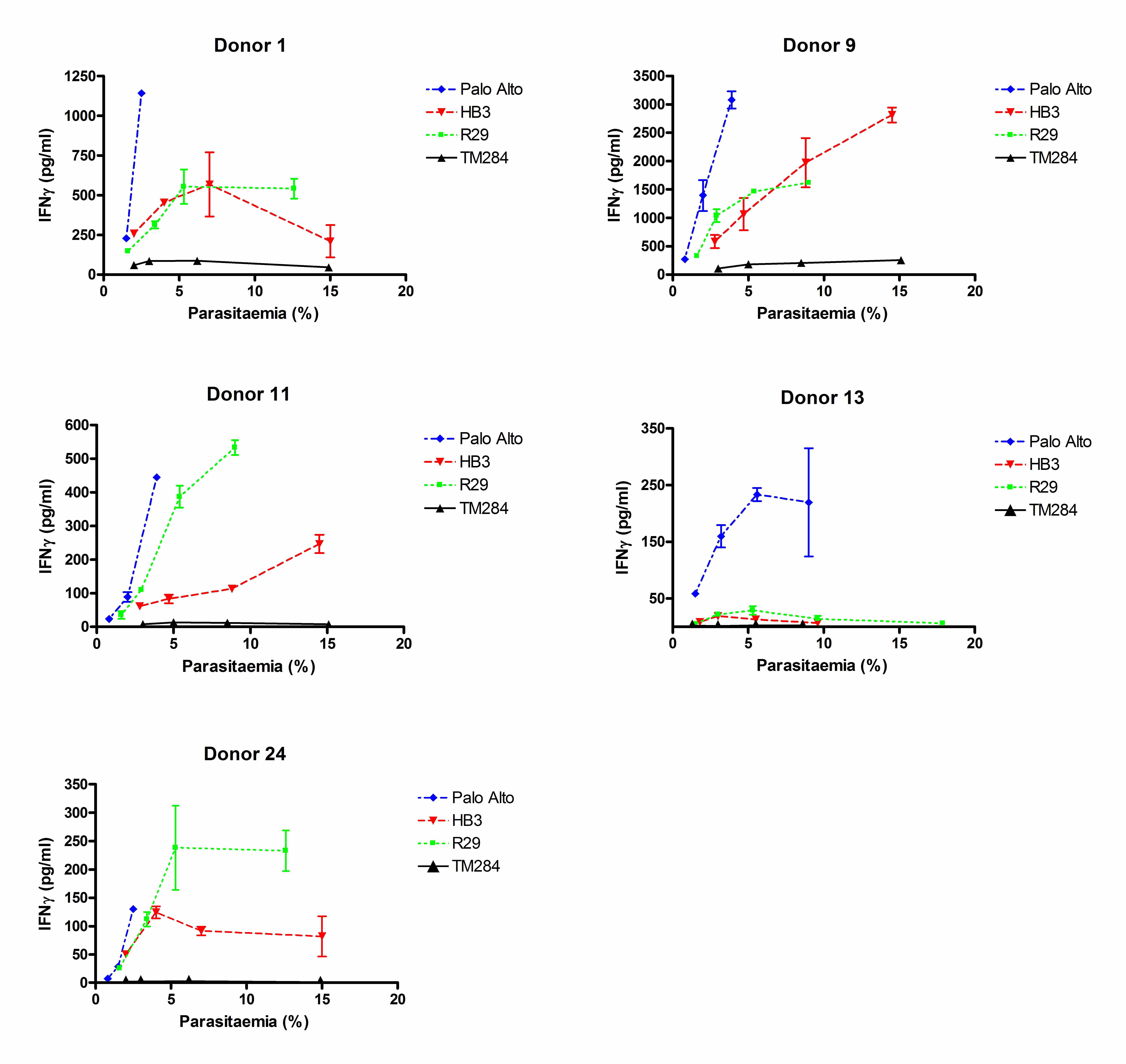


Supplementary figure 1





Supplementary figure 2


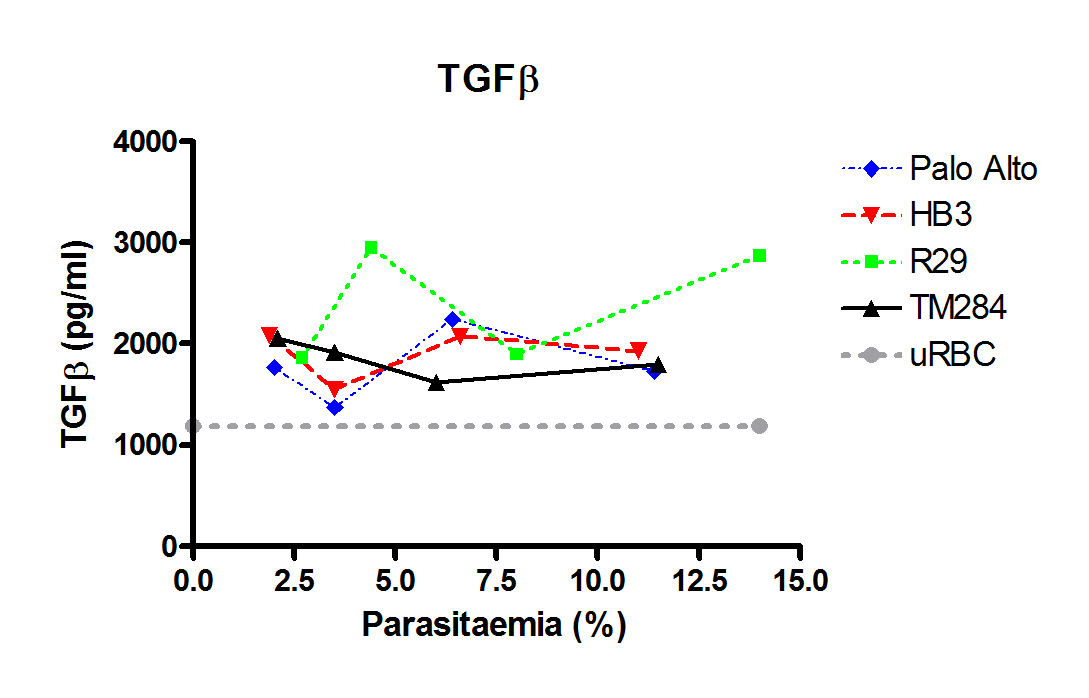


Supplementary figure 3


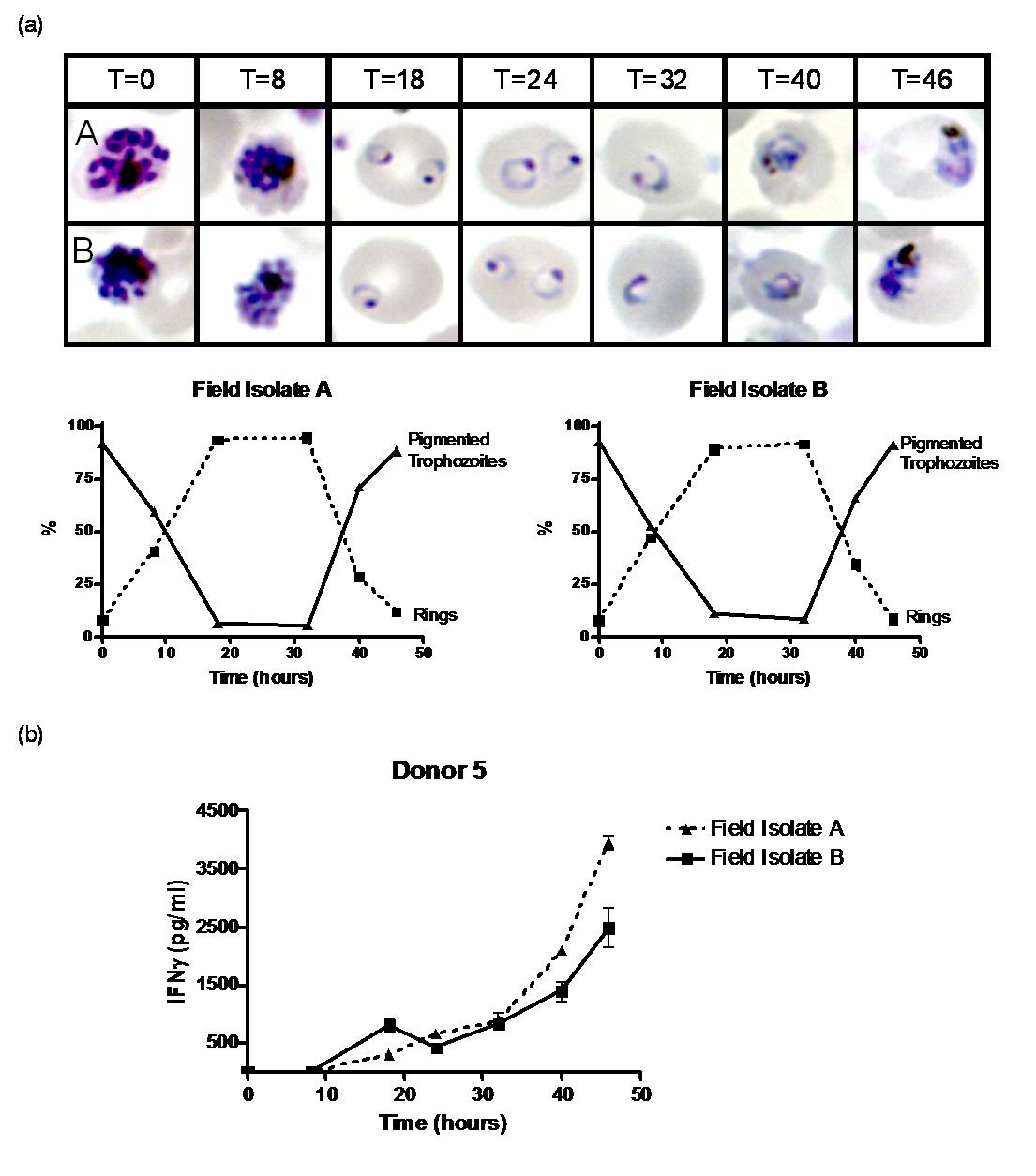


Supplementary figure 4

Supplement: Supplementary file 1 [file pim0032-0512-SD1.doc]
