# Supplementary material for: Strain variation in early innate cytokine induction by Plasmodium falciparum
Source: Parasite Immunol. 2010 Jul;32(7):512–27. doi: 10.1111/j.1365-3024.2010.01225.x (PMC2941733; doi:10.1111/j.1365-3024.2010.01225.x)
Supplement: Supplementary file 2 [file pim0032-0512-SD2.doc]

**Supplementary Information, Corrigan and Rowe.**

**Supplementary Table 1. Output of GLM regression analysis assessing the contributions of PBMC donor, parasite strain and parasitaemia on production of pro-inflammatory cytokines after 24 hours co-culture of *P. falciparum* with human PBMCs.**

| **Cytokine** | **Factor** | **Df** | **F** | **P** | **R^2^ (%)** |
| --- | --- | --- | --- | --- | --- |
| GM-CSF | Strain | 3 | 27.83 | **<0.001** | 73.59 |
|  | Donor | 7 | 30.92 | **<0.001** |  |
|  | Parasitaemia | 1 | 100.6 | **<0.001** |  |
|  | Error | 118 |  | |  |
| IL-1β | Strain | 3 | 30.02 | **<0.001** | 62.62 |
|  | Donor | 7 | 7.53 | **<0.001** |  |
|  | Parasitaemia | 1 | 113.79 | **<0.001** |  |
|  | Error | 118 |  | |  |
| IL-6 | Strain | 3 | 28.19 | **<0.001** | 63.65 |
|  | Donor | 7 | 12.52 | **<0.001** |  |
|  | Parasitaemia | 1 | 94.83 | **<0.001** |  |
|  | Error | 118 |  | |  |
| TNFα | Strain | 3 | 27.68 | **<0.001** | 59.65 |
|  | Donor | 7 | 12.02 | **<0.001** |  |
|  | Parasitaemia | 1 | 58.76 | **<0.001** |  |
|  | Error | 118 |  | |  |

Df: Degrees of freedom

**Supplementary Figure Legends.**

**Supplementary Figure 1. IFNγ levels after 24 hours co-culture of *P. falciparum* infected erythrocytes with human PBMCs for five additional donors.** 5x10^5^ human PBMCs were co-cultured with 2.5x10^7^ erythrocytes infected with 1 of 4 laboratory strains of *P. falciparum* (TM284, R29, HB3, Palo Alto) across a range of parasitaemia for 24 hours. The IFNγ level in the culture supernatants were measured by ELISA. Across all 11 donors (the other six donors are shown in Figure 1) the IFNγ response after PBMC stimulation with *P. falciparum* was significantly dependent on parasite strain (F_3,178_=48.49, P<0.001). Error bars (where shown) show the standard error about the mean of at least duplicate wells for each data point.

**Supplementary Figure 2. Cytokine production after 24 hours co-culture of *P. falciparum* infected erythrocytes with human PBMCs for seven additional donors.** 5x10^5^ human PBMCs were stimulated with 2.5x10^7^ erythrocytes infected with 1 of 4 laboratory strains of *P. falciparum* (TM284, R29, HB3, Palo Alto) across a range of parasitaemia for 24 hours. IFNγ, TNFα, IL-6, GM-CSF, IL-1β and IL-10 levels in the culture supernatants from eight donors were measured by multiplex bead assay. Data from seven donors are shown (see Figure 2 for data from an additional donor). Across all donors, the cytokine response after PBMC stimulation with *P. falciparum* is significantly dependent on parasite strain (P<0.001 for each cytokine, see Supplementary Table 1).

**Supplementary Figure 3. Total TGFβ levels after 24 hours co-culture of *P. falciparum* infected erythrocytes with human PBMCs.**  5x10^5^ human PBMCs were co-cultured with 2.5x10^7^ erythrocytes infected with 1 of 4 laboratory strains of *P. falciparum* (TM284, R29, HB3, Palo Alto) across a range of parasitaemia for 24 hours. Total TGFβ levels in the culture supernatants were measured by multiplex bead assay. The results shown are from donor 3, and the five other donors tested showed similar patterns. Across all six donors the variance in TGFβ levels was not significantly affected by parasite strain (F_3,84_=1.24, P=0.3) or parasitaemia (F_1,84_=0.38, P=0.539), but was significantly affected by PBMC donor (F_5,84_=11.18, P<0.001).

**Supplementary Figure 4. IFNγ produced by human PBMCs after 46 hours co-culture with two *P. falciparum* field isolates from African patients.** 2.5 x 10^7^ erythrocytes infected with either field isolate A or B at 5% parasitaemia were co-cultured with 5x10^5^ human PBMCs from a single donor over 46 hours. (a) Parasite maturity was assessed every 6-8 hours throughout the time course from Giemsa stained thin films. The parasitaemia and percentage rings and pigmented trophozoites/schizonts were counted at each time point. (b) The IFNγ level in culture supernatants was measured by ELISA at each time point. Error bars show the standard error from duplicate wells.
